# Urinary biomarkers indicate pediatric renal injury among rural farming communities in Sri Lanka

**DOI:** 10.1038/s41598-022-10874-w

**Published:** 2022-05-16

**Authors:** T. D. K. S. C. Gunasekara, P. Mangala C. S. De Silva, E. M. D. V. Ekanayake, W. A. K. G. Thakshila, R. A. I. Pinipa, P. M. M. A. Sandamini, S. D. Gunarathna, E. P. S. Chandana, S. S. Jayasinghe, C. Herath, Sisira Siribaddana, Nishad Jayasundara

**Affiliations:** 1 Department of Zoology, Faculty of Science, University of Ruhuna, Matara, 81000 Sri Lanka; 2 Department of Biomedical Sciences, School of Medicine and Health Sciences, University of North Dakota, Grand Forks, ND 58203 USA; 3 Department of Biosystems Technology, Faculty of Technology, University of Ruhuna, Matara, 81000 Sri Lanka; 4 Department of Pharmacology, Faculty of Medicine, University of Ruhuna, Galle, 80000 Sri Lanka; 5 Department of Nephrology, Sri Jayewardenepura General Hospital, Colombo, 10100 Sri Lanka; 6 Department of Medicine, Faculty of Medical and Allied Sciences, Rajarata University, Saliyapura, 50008 Sri Lanka; 7 The Nicholas School of the Environment, Duke University, Durham, NC 27708 USA

**Keywords:** Biomarkers, Nephrology

## Abstract

Pediatric renal injury is an emerging health concern in communities affected by chronic kidney disease of uncertain etiology (CKDu). Early detection of susceptibilities through highly sensitive and specific biomarkers can lead to effective therapeutic and preventive interventions against renal diseases. Here, we aimed to investigate the utility of kidney injury molecule (KIM-1) and neutrophil gelatinase-associated lipocalin (NGAL) in early detection of renal abnormalities in selected pediatric communities in Sri Lanka. The study areas were stratified as CKDu endemic, emerging, and non-endemic based on the prevalence of CKDu, and a total of 804 school students (10–18 years of age) participated in the study. The median (IQR) urinary KIM-1 levels of the participants were 0.193 (0.026–0.338), 0.082 (0.001–0.220) and 0.040 (0.003–0.242) ng/mgCr for CKDu endemic, emerging and non-endemic regions respectively. Participants from CKDu endemic regions reported elevated (*p* < 0.0001) urinary KIM-1 expression compared to those from the other regions. The median (IQR) NGAL levels in participants from CKDu endemic (2.969; 1.833–5.641), emerging (3.374; 1.766–6.103), and non-endemic (3.345; 1.742–5.128 ng/mgCr) regions showed no significant difference. Also, urinary albumin-creatinine ratio (UACR) showed no significant differences across gender or residency. The prevalence of albuminuria was 1–2% in the locations irrespective of CKDu burden. Albuminuric participants reported higher (*p* < 0.05) urinary KIM-1 levels in comparison to normoalbuminuric participants. Significantly elevated urinary KIM-1 expression in a pediatric population from CKDu affected regions, especially in the presence of albuminuria, may indicate low-grade early renal damage supporting the utility of KIM-1 as a quantifiable biomarker.

## Introduction

Chronic kidney disease (CKD) is pathophysiologically complex and asymptomatic, especially at the early stages. Hence, it is often diagnosed late, and the associated global burden is underreported. According to global approximations, 850 million people are known to be affected by renal diseases including CKD, acute kidney injury (AKI), and even more critical complications, requiring renal replacement therapy or dialysis^1^. Annually kidney diseases account for more than 5 million deaths, and remain the fifth leading cause of death globally^2^.

CKD of uncertain aetiology (CKDu) is an emerging global health problem affecting socio-economically disadvantaged rural farming communities in Central America and South Asia including Sri Lanka^3^. It occurs in the absence of conventional risk factors of CKD such as diabetes mellitus, hypertension and glomerular diseases, and the exact aetiology remains unresolved^4^. CKDu is predominant among agricultural communities across global hotspots. Due to the close association with agricultural lifestyle, this disease is also referred to as chronic interstitial nephritis among agricultural communities (CINAC)^5^. Most risk factors are environmental where nephrotoxic contaminants (e.g., pesticides and heavy metals) and heat stress, have been identified to play a role^6^.

Although in global hotspots a majority of CKDu cases represents adult males^7^, a few studies have demonstrated the prevalence of certain renal abnormalities among school-age pediatric populations in CKDu affected areas in Sri Lanka^8^ and Mesoamerica^9^. A recent analysis of CKD/ CKDu statistics (from 2003 to 2017) in the CKDu endemic Anuradhapura and Polonnaruwa districts in the North Central Province in Sri Lanka revealed that 0.25%, 1.59%, and 2.12% of the cases were below 10 years, 10–20 years, and 20–30 years of age respectively, where 73% of the CKDu patients were over the age of 50^10^. It is possible that the onset of CKDu may occur in the early stages of life due to exposure to environmental risk factors^11^.

Both CKD and CKDu are progressive nephropathies that remain asymptomatic until the late stages^12^, and the disease is often diagnosed in late adulthood. Given the potential early life onset with CKDu, screening approaches with robust biomarkers to identify early renal injury is of utmost importance in the management of CKD/ CKDu, particularly among vulnerable communities.

Currently, serum creatinine, proteinuria and blood urea nitrogen serve as the main diagnostic tools in the clinical diagnosis of renal injury. However, they are less optimal in terms of sensitivity and specificity, to detect kidney disease early^13^. A wide spectrum of emerging biomarkers appears to be potentially advantageous in the early detection of renal injury and disease susceptibilities. Several biomarkers including, Kidney Injury Molecule-1 (KIM-1), Neutrophil Gelatinase-Associated Lipocalin (NGAL), N-acetyl-beta-D-glucosaminidase (NAG), Cystatin-C (CysC), clusterin (CLU) and osteopontin (OPN) are approved as biomarkers to detect renal tubular injury in phase 1 trials, by the United States Food and Drug Administration (FDA-USA)^14^. Current findings in diverse clinical settings have provided scientific evidence on the improved diagnostic efficacy of these biomarkers over the conventional markers^3^.

The utility of KIM-1 and NGAL has been assessed in both adult and pediatric populations in Sri Lanka and Mesoamerica^3^. KIM-1 is a type I transmembrane protein with extracellular, transmembrane, and intracellular (cytoplasmic) domains^15^. Based on the variability in the cytoplasmic domain, two homologs of KIM-1 have been identified in humans^16^. The KIM-1a variant is predominantly expressed in the liver^17^, while the KIM-1b variant is primarily expressed in the kidney. KIM-1 is mainly expressed in the apical membrane of tubular epithelial cells immediately following renal injury^18^. The extracellular domain of KIM-1 undergoes cleavage, releasing soluble KIM-1 into the extracellular space making it available in the urine^19^. In a healthy kidney, KIM-1 may be expressed at very low levels^17,20^ and its expression is upregulated in renal damage leading to higher concentrations of KIM-1 in urine^21^. Several studies indicate that urinary KIM-1 is a promising diagnostic and prognostic biomarker in CKD and AKI^17,20^.

NGAL is a member of the lipocalins superfamily, specialized in binding and transporting small hydrophobic molecules^22^. Human NGAL was identified and isolated from secondary granules of neutrophils^23^. In addition to the kidney, NGAL gene expression occurs in a variety of human tissues such as liver, trachea, lung, bone marrow, uterus, prostate, salivary gland, stomach, and colon contributing appearance of NGAL mainly in circulation at low levels^24^. NGAL in circulation is filtered by the glomeruli and reabsorbed at the proximal tubule. NGAL is secreted by the thick ascending limb of the renal tubule accounting for the presence of NGAL in urine at low concentrations. In proximal tubular injury, NGAL synthesis is increased and reabsorption may be decreased resulting in elevated NGAL levels in urine^25^. Further, distal tubular injury also causes increased synthesis of NGAL and its secretion also contributing to high NGAL levels in urine^26^. Hence urinary NGAL is considered a promising marker of renal injury and its diagnostic and prognostic potential is evident in many studies^27,28^.

Considering the importance of the detection of compromised renal health as earlier as possible, in the pediatric populations, here we investigated the levels of KIM-1 and NGAL in a pediatric population in Sri Lanka. The objective of our study was to examine the utility of KIM-1 and NGAL as biomarkers of renal abnormalities and establish their potential in the early identification of renal dysfunction in pediatric communities at high risk of CKDu.

## Materials and methods

### Establishment of study groups

**Figure 1:**
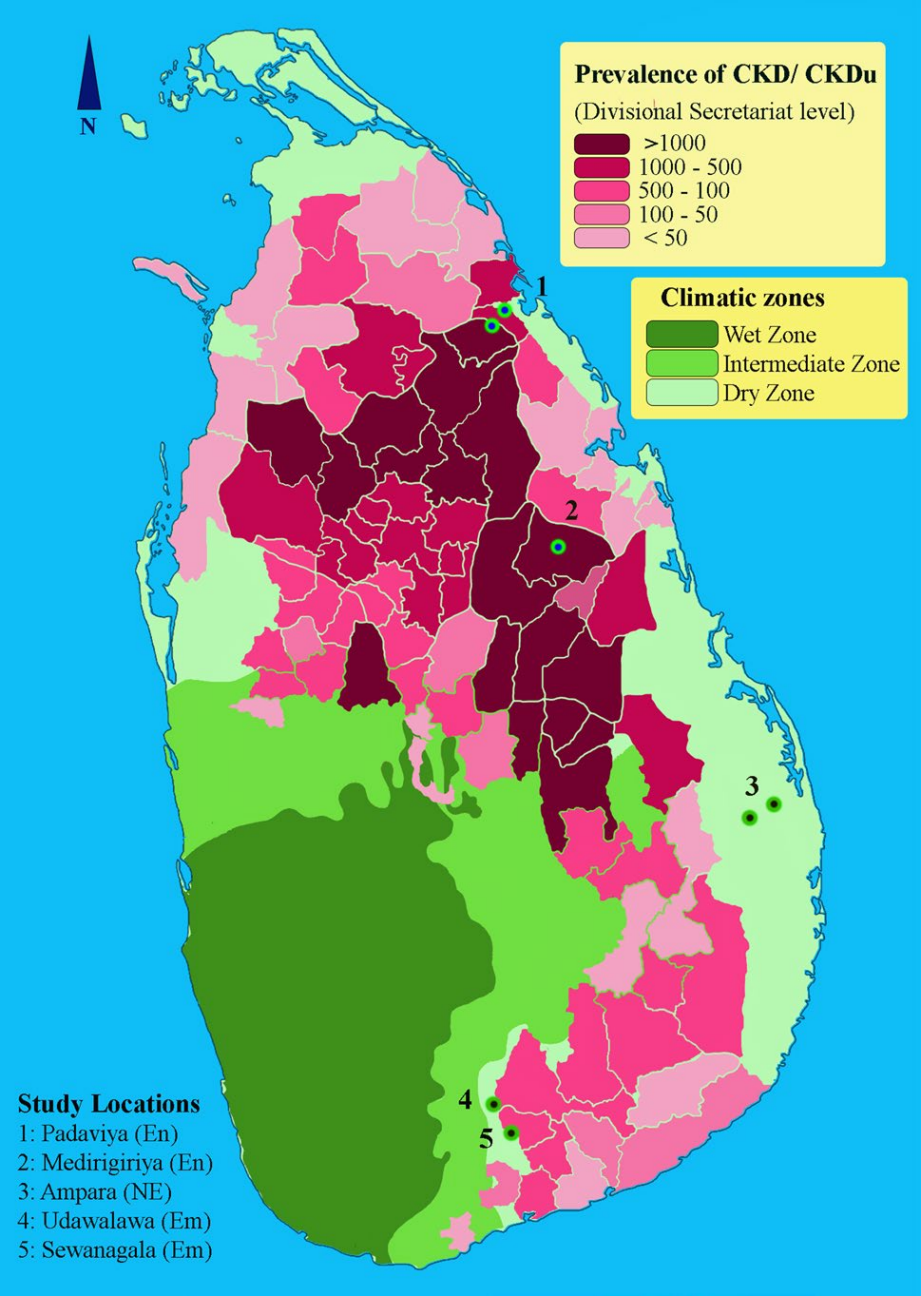
The selected study locations representing CKDu endemic (En), emerging (Em) and non-endemic (NE) regions, with respect to Burden of CKDu and climatic zones in Sri Lanka. The prevalence of CKDu is expressed as the number of CKD/CKDu cases at Divisional Secretariat levels, based on hospital records as per the analysis of Ranasinghe et al.^10^. The map was created using QGIS 3.22.4 (https://www.qgis.org/en/site/) and Adobe Photoshop (https://www.adobe.com/sg/products/photoshop.html).

For the assessment of renal biomarker distributions, we selected school students with a multi-stage stratified proportionate random sampling approach. Based on the degree of CKDu prevalence at Divisional Secretariat level in Sri Lanka^10,29^, regions were categorized into three strata; CKDu endemic, CKDu emerging and non-endemic. We defined CKDu endemic areas as regions with highest prevalence of clinically confirmed CKDu with well established adult CKDu patient groups. Regions with comparatively low incidence of clinically diagnosed CKDu cases with evidence of increasing CKDu prevalence were considered as CKDu emerging regions. We defined CKDu non-endemic regions where very low incidence of CKDu is reported or the prevalence of CKDu is not evident. Most of the CKDu endemic and emerging regions were in the dry zone, while wet and intermediate zones occupied majority of CKDu non-endemic regions. Divisional secretariats with similar climatic and socioeconomic determinants were stratified according to the burden of CKDu, as CKDu endemic, emerging and non-endemic, and Divisional secretariats were selected using systematic random sampling, proportionately to the residential population for the study. Padaviya and Medirigiriya Divisional secretariats located in North Central Province, were selected as CKDu endemic. Embilipitiya (Sabaragamuwa Province) and Sevanagala (Uva Province) divisional secretariats were selected as CKDu emerging and Ampara Divisional secretariat (Eastern Province) was selected as non-endemic. The selected study locations are shown in Fig. 1 with respect to the prevalence of CKD/CKDu in Sri Lanka.

Schools for the study were selected with multi-stage cluster sampling from the government schools in the selected Divisional Secretariats. We identified the study participants based on random selection from the grades six to thirteen in the selected schools. Nonresidential students and the students with duration of residency less than eight years in the respective areas were excluded from participation. The students fulfilling the following inclusion criteria were enrolled for the study.

- Girls and boys aged between 10 and 18 years at the time of enrollment.
- Assent of the participants and the written consent from parents for participation, medical examination, donation of samples and long-term storage, and to produce records on medical history and current medications.
- Willingness to be contacted further.

Based on the formula; n = [(z^2^) P (1–P)]/d^2^, the minimum sample size was calculated. The standard normal variate (z^2^) was taken as 2.58 at 1% type 1 error (*p* < 0.01) and the absolute error (d) was assumed to be 5% (d = 0.05)^30^. As interpreted by moderately and highly elevated UACR among the children in CKDu endemic regions in Sri Lanka, the prevalence of abnormal renal function (P), was taken as 8.7% (*p* = 0.087) based on the most recent pediatric study conducted by Agampodi et al. in 2018^8^. Accordingly, the estimated minimum sample size was 212 for the CKDu endemic regions where the highest incidence of renal abnormalities could be expected. The same sample size was taken from CKDu emerging regions and 50% of endemic sample size (N = 106) was taken from CKDu non-prevalent regions for comparison. A multi-stage stratified proportionate random sampling approach was adopted for selection of schools and children in CKDu endemic, emerging and non-endemic regions. A total of 327 children (150 boys and 177 girls) representing CKDu endemic regions, and 313 children (140 boys and 173 girls) representing CKDu emerging regions, and 164 children (86 boys and 78 girls) representing non-endemic regions, participated in the study.

### Sample and data management

An interviewer-administered structured questionnaire was used for the collection of demographic data, and the details on medical history, lifestyle habits, family history of diseases, current health status, and medications. The height and weight of the participants were measured using a stadiometer.

Early morning first voided urine sample was obtained from each participant into a sterile container for the analysis. Samples were collected between 6–8 am and brought to the collection points (schools) at room temperature and stored at 2–8 °C until centrifugation. The samples were centrifuged at 1000 RCF for 15 min at 4 °C and the supernatant was isolated. The supernatant was stored at − 80 °C for the assessment of renal injury biomarkers^31^.

### Assessment of renal biomarkers

KIM-1 and NGAL were assessed using Enzyme-Linked Immunosorbent Assay (ELISA) kits (Cusabio Technology LLC, Wuhan, China). As specified by the manufacturer, inter-assay precision and intra-assay precision values for the KIM-1 and NGAL ELISA kits were CV% < 10% and CV% < 8%, respectively^31^. Urine samples were analyzed for creatinine and microalbumin using an automated biochemistry analyzer (HumaStar 100; Human mbH, Wiesbaden, Germany). 

### Data and statistical analysis

Baseline KIM-1 and NGAL concentrations in each urine sample were normalized to their creatinine content and expressed as adjusted concentrations^32^. The data were partitioned according to gender and stratified according to age before the analysis. The Shapiro–Wilk test was used to determine the distribution pattern of creatinine-adjusted urinary biomarker concentrations^33,34^. The distribution of data deviated from a normal distribution towards a log normal distribution, hence a nonparametric statistical approach was adopted. Kruskal–Wallis one-way analysis followed by Dunn’s multiple comparison test was used for comparison of creatinine-adjusted urinary biomarker levels of children in the same age range, from CKDu endemic, emerging and non-endemic regions^33,34^. Mann–Whitney U test was used for comparison of biomarker levels across gender within the study groups^33^. Statistical analysis was performed using GraphPad Prism 9.3 (GraphPad software LLC, San Diego, USA) and IBM SPSS Statistics 26.0 (IBM INC., New York, USA).

### Ethical considerations

Ethics Review Committee of the Faculty of Medicine, University of Ruhuna, Galle, Sri Lanka approved the study (reference no: 2020.P.124; 20 November 2020). Before participation, informed written consent of the parents and the assent of the study participants were obtained.

### Ethical approval

The study was conducted according to the guidelines of the Declaration of Helsinki, and approved by the Ethics Review Committee of the Faculty of Medicine, University of Ruhuna, Galle, Sri Lanka (reference no: 2020.P.124; Date: 29.01.2021).

## Results

### Sociodemographic characteristics of participants

A total of 804 school students between 10–18 years of age were recruited to participate in the study and the main sociodemographic and clinical characteristics of the participants are given in Table 1.

**Table 1:** Sociodemographic and clinical characteristics of the study participants.

Criterion	Endemic (N = 327)		Emerging (N = 313)		Non-endemic (N = 164)	
	Boys	Girls	Boys	Girls	Boys	Girls
No. of children	150	177	140	173	86	78
**Age (years)** Median (IQR)	14.4 (13.6–15.1)	14.6 (13.9–15.5)	14.6 (13.9–15.3)	14.4 (13.6–15.2)	13.6 (12.7–14.7)	13.8 12.6–15.0)
**BMI (kg/m**^**2**^**)** Median (IQR)	17.8 (15.6–19.9)	18.3 (17.0–20.4)	16.8 (15.3–19.1)	17.5 (15.4–20.2)	16.9 (15.3–19.1)	19.1 (16.5–22.9)
**Renal disorders**						
Renal stones	2 (0.6%)	0	1 (0.3%)	0	4 (2.4%)	0
Pain when urinating	0	0	1 (0.3%)	0	4 (2.4%)	1 (0.6%)
infections	0	0	0	0	0	1 (0.6%)
**Other diseases**						
Asthma	0	0	0	0	5 (3.0%)	2 (1.2%)
Dental fluorosis	4 (1.2%)	3 (0.9%)	0	0	0	0
**Family history CKD/CKDu**	46 (14.1%)	37 (11.3%)	0^‡^	7^‡^ (2.2%)	2^‡^ (1.2%)	6^‡^ (3.7%)
**Parents’ occupation as farmers**	120 (36.7%)	135 (41.3%)	57^‡^ (18.2%)	70^‡^ (22.4%)	12^‡ ⁑^ (7.3%)	11^‡ ⁑^ (6.7%)

The occurrence of renal disorders, other diseases, family history of chronic kidney disease of uncertain etiology (CKDu) and parents’ involvement in farming is given as the number of children and as a percentage of the total size of respective group. Statistical significance between proportions is expressed for boys and girls in CKDu emerging and non-endemic areas compared to their counterparts from other groups according to Chi-squared test; ^**‡**^denotes comparison with endemic group (*p* < 0.05) and ^⁑^denotes comparison with emerging group (*p* < 0.05). *BMI-* body mass index, and *IQR-* inter quartile range. Age is given to the date of sample collection.

No significant differences in BMI of the children were observed across gender or the region. However, a very low prevalence of renal stones was detected among the participants. Rendering the high burden of CKDu, among the participants in endemic areas, the history of a family member diagnosed with CKDu was significantly higher than that of the participants from the other regions. Further, the involvement of parents of the participants in farming was significantly high in CKDu endemic and emerging regions compared to the CKDu non-endemic regions.

### Urinary KIM-1, NGAL and ACR levels in children

The experimental inter-assay and intra-assay precision coefficient of variation was 4.16% and 3.25% for KIM-1 and 5.28% and 4.08% for NGAL. The distributions of creatinine-adjusted urinary KIM-1 and NGAL levels in children were assessed across the three study groups from CKDu endemic, emerging, and non-endemic regions in Sri Lanka (Table 2).

**Table 2:** Levels of KIM-1, NGAL, and ACR in children from CKDu endemic, emerging, and non-endemic regions.

Biomarker	CKDu endemicity in residential area			Comparison
	Endemic (En) (N = 327)	Emerging (Em) (N = 313)	Non-endemic (NE) (N = 164)	
**KIM-1(ng/mgCr)** Median (IQR)	0.193 (0.026–0.388)	0.082 (0.001–0.220)	0.040 (0.003–0.242)	**En-Em: *p* < 0.0001** **En-NE: *p* < 0.0001** Em-NE: *p* = 0.478
**NGAL (ng/mgCr)** Median (IQR)	2.969 (1.833–5.641)	3.374 (1.766–6.013)	3.345 (1.742–5.128)	En-Em: *p* = 0.719 En-NE: *p* > 0.999 Em-NE: *p* = 0.651
**ACR (mg/g)** Median (IQR)	2.435 (1.536–4.188)	2.459 (1.494–4.015)	2.480 (1.518–4.122)	En-Em: *p* > 0.999 En-NE: *p* = 0.082 Em-NE: *p* = 0.439

significant comparisons are shown in bold.

Biomarker levels for children in the three study groups are expressed as median with inter quartile range (IQR) and inter-group comparison is expressed in terms of Kruskal–Wallis one-way analysis followed by Dunn’s multiple comparison test.

*KIM-1* kidney injury molecule-1, *NGAL* neutrophil gelatinase-associated lipocalin, *ACR* albumin creatinine ratio, *CKDu* burden in the study regions, *EN* endemic, *Em* emerging and *NE* non-endemic.

**Figure 2:**
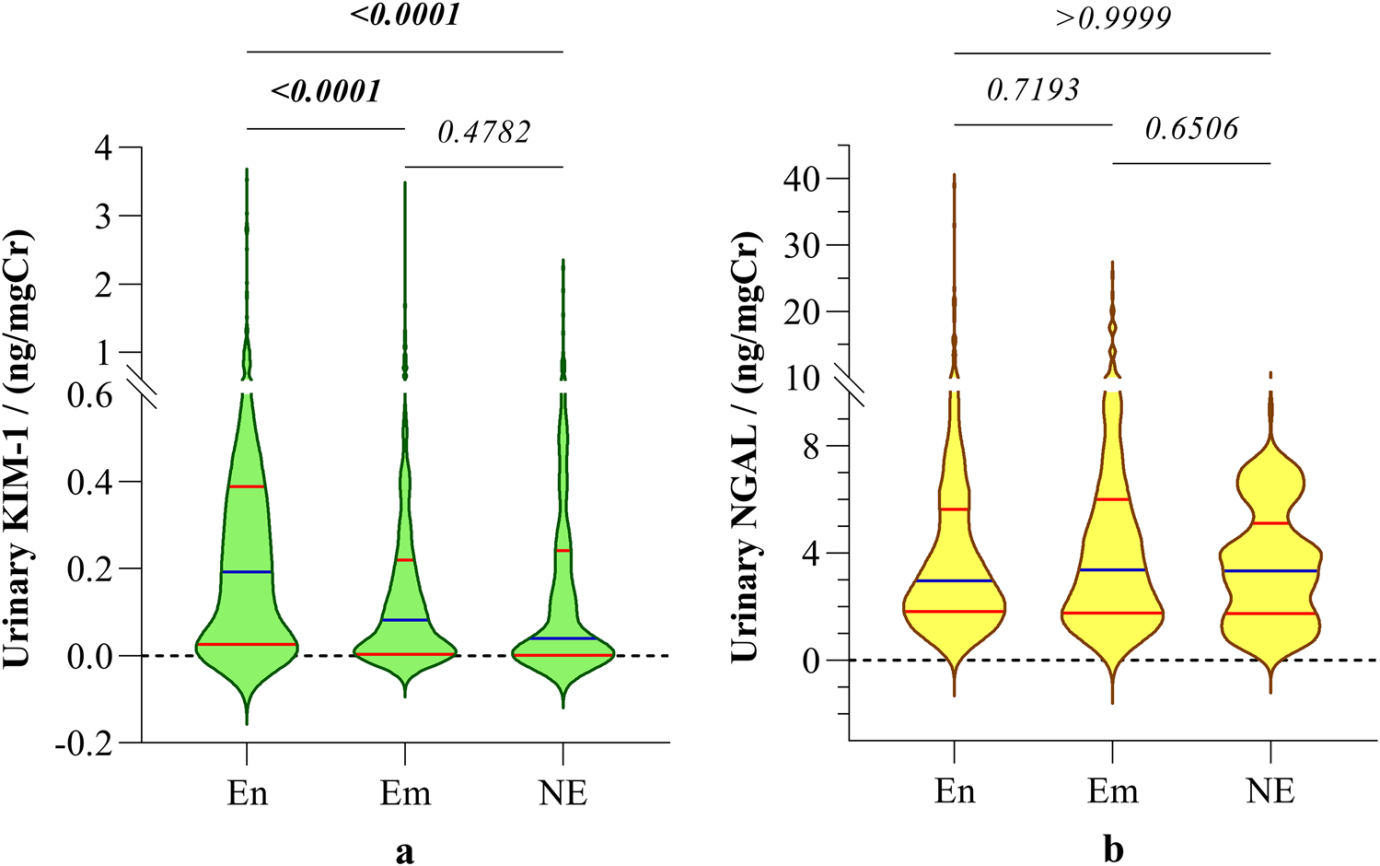
Distribution of urinary biomarkers (**a**) KIM-1 and (**b**) NGAL in children from CKDu endemic, emerging and non-endemic regions irrespective of gender. Graphs illustrate the median and interquartile range. Inter-group comparison is expressed in terms of Kruskal–Wallis one-way analysis followed by Dunn’s multiple comparison test. The study groups: En: endemic, Em: emerging and NE: non-endemic regions. KIM-1: Kidney injury molecule-1; NGAL: Neutrophil gelatinase-associated lipocalin.

Irrespective of gender, urinary KIM-1 levels in children from CKDu endemic areas were significantly higher (*p* < 0.0001), compared to the children from CKDu emerging and nonendemic areas. On the contrary, urinary NGAL levels in children did not demonstrate significant difference across endemic emerging and non-endemic regions (Table 2). The distribution of urinary biomarkers is shown in Fig. 2.

**Figure 3:**
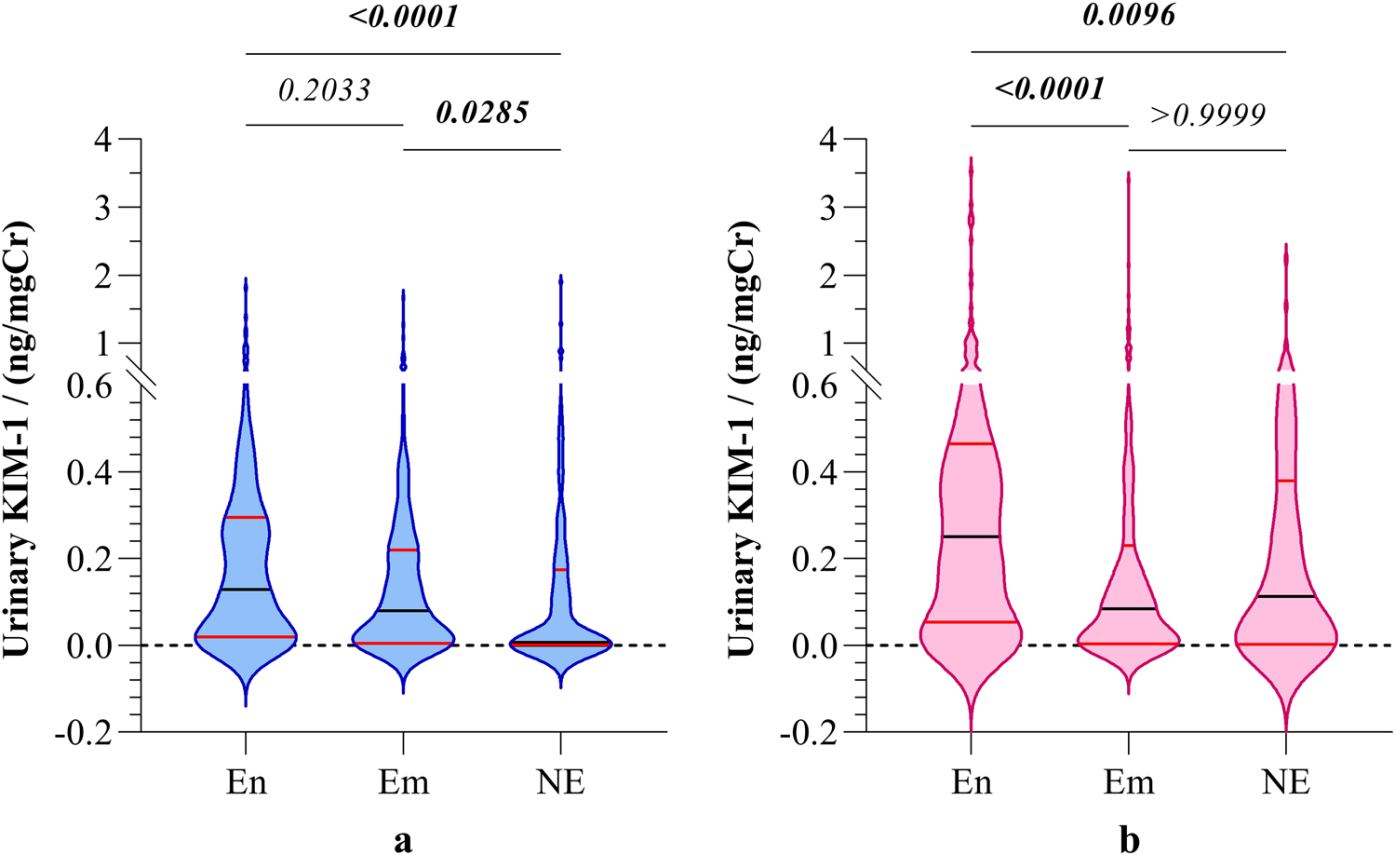
Gender-stratified distribution of urinary KIM-1 in (**a**) boys and (**b**) girls from CKDu endemic, emerging, and non-endemic regions. Graphs illustrate the median and interquartile range. Inter-group comparison is expressed in terms of Kruskal–Wallis one-way analysis followed by Dunn’s multiple comparison test. The study groups: En: endemic, Em: emerging, and NE: non-endemic regions. KIM-1: Kidney injury molecule-1.

Further analysis of biomarker levels of the boys and girls separately rendered significant difference of KIM-1 levels in participants among the three study groups. Both girls and boys in endemic regions reported significantly higher urinary KIM-1 levels compared to their counterparts in CKDu non-endemic regions (Fig. 3).

**Figure 4:**
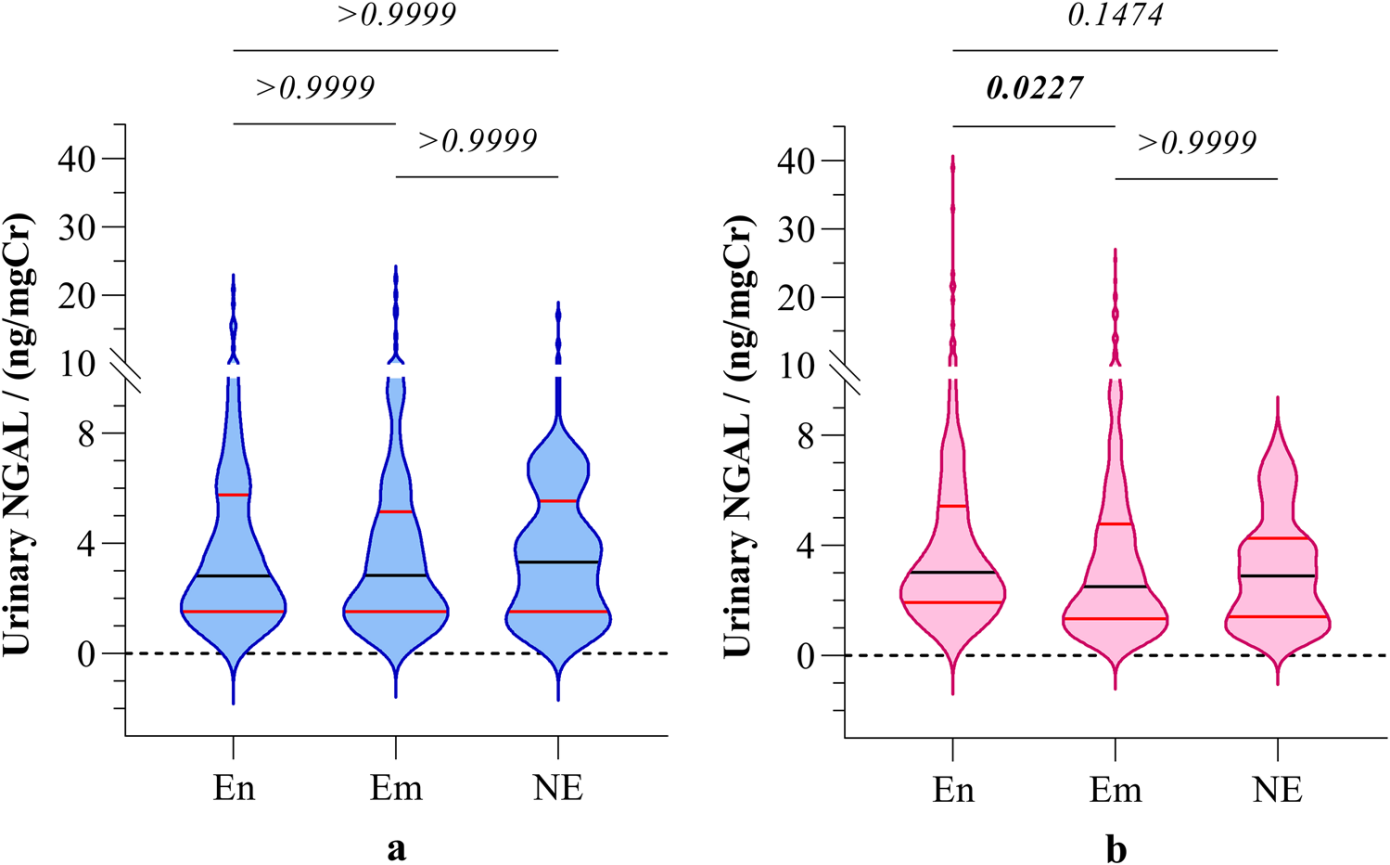
Gender-stratified distribution of urinary NGAL (**a**) boys and (**b**) girls from CKDu endemic, emerging, and non-endemic regions. Graphs illustrate the median and interquartile range. Inter-group comparison is expressed in terms of Kruskal–Wallis one-way analysis followed by Dunn’s multiple comparison test. The study groups: En: endemic, Em: emerging, and NE: non-endemic regions. NGAL: neutrophil gelatinase associated lipocalin.

However, urinary NGAL levels of the participants showed no significant difference among boys in the three study groups. In contrast, girls in CKDu endemic regions showed significantly elevated (*p* < 0.05) NGAL level compared to the girls in emerging regions, but not with the girls in non-endemic regions. (Fig. 4).

**Figure 5:**
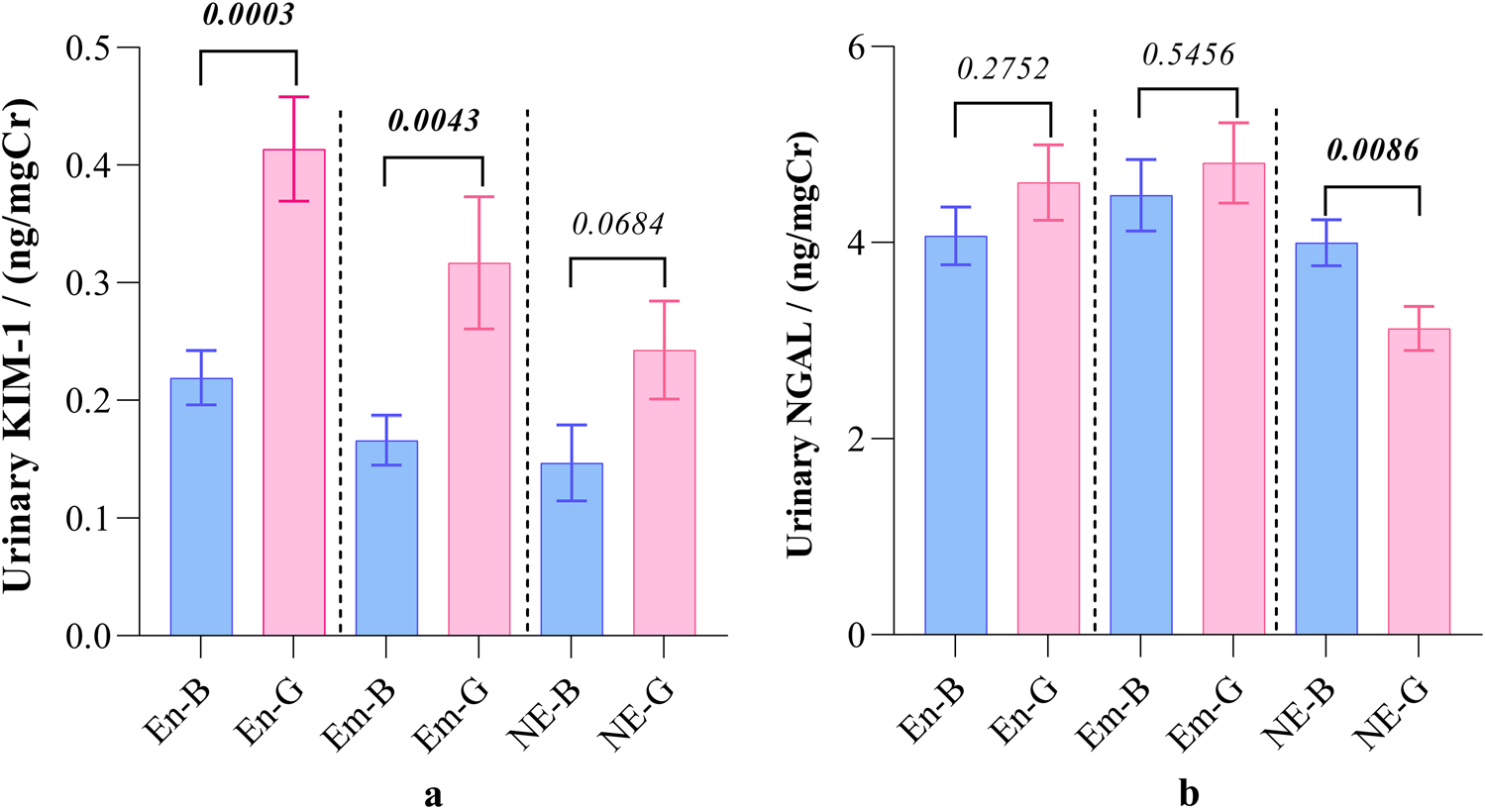
Expression of biomarkers in urine. (**a**) KIM-1 and (**b**) NGAL across gender in the three study groups. Graphs represent mean with standard error of mean (SEM). Comparison between the boys and girls within the same group is expressed in terms of Mann–Whitney U test. The study groups: En: endemic, Em: emerging, and NE: non-endemic regions. B: boys and G: girls. KIM-1: kidney injury molecule and NGAL: neutrophil gelatinase associated lipocalin.

In each study group, girls reported elevated urinary KIM-1 levels compared to the boys. The difference was significant (*p* < 0.01) in CKDu endemic and emerging groups, but not in the non-endemic group. On the contrary, urinary NGAL levels showed no significant variation between the two genders in CKDu endemic and emerging groups. However, girls in non-endemic regions reported significantly low (*p* < 0.01) urinary NGAL level compared to the boys (Fig. 5).

**Figure 6:**
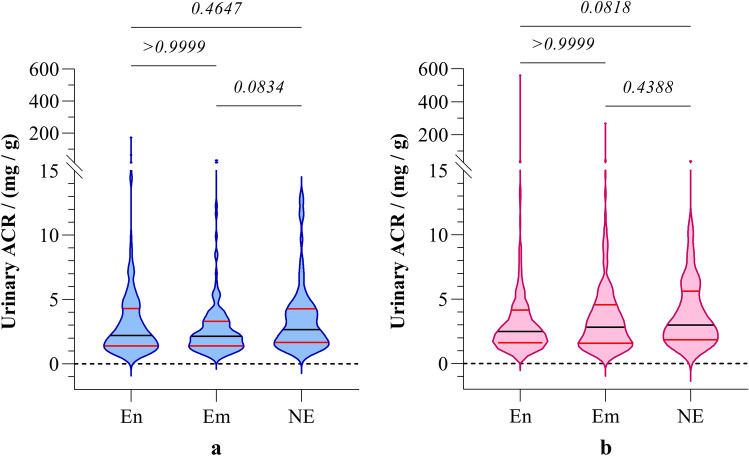
Gender stratified distribution of urinary ACR in (**a**) boys and (**b**) girls from CKDu endemic, emerging, and non-endemic regions. Graphs illustrate the median and interquartile range. Inter-group comparison is expressed in terms of Kruskal–Wallis one-way analysis followed by Dunn’s multiple comparison test. The study groups: En: endemic, Em: emerging, and NE: non-endemic regions. ACR: albumin to creatinine ratio.

No significant differences in ACR values were observed between CKDu endemic, emerging, and non-endemic regions (Table 2). Stratification of ACR across the gender also showed no significant differences in ACR among the three study groups (Fig. 6).

Further, urinary ACR showed no significant variation between the boys and girls within the same study group (data are not shown). In contrast to the novel biomarkers, urinary ACR appears to be less sensitive in indicating minor alterations in renal function.

### Assessment of the status of renal function in age groups

#### KIM-1 and NGAL levels

As per the reference intervals given in our previous study (50th quantile at 95% CI) in school children of Sri Lanka^35^, the incidence of elevated biomarker levels above the given reference intervals in each age group is shown in Tables 3 and 4.

**Table 3:** The incidence of elevated expression of of urinary KIM-1 in children from CKDu endemic, emerging, and non-endemic regions in Sri Lanka.

Age group	RI^35^ ng/mgCr	Endemic		Emerging		Non-endemic	
		N_T_	KIM > RI N (%)	N_T_	KIM > RI N (%)	N_T_	KIM > RI N (%)
**Boys**							
10.0–12.9	0.097	4	2 (0.61)^**†**^	0	0 (0.00)	31	12 (7.32)
13.0–13.9	0.094	55	28 (8.56)	39	19 (6.07)	22	8 (4.88)
14.0–14.9	0.080	46	30 (9.17)^**†**^	42	19 (6.07)	18	4 (2.44)
15.0–15.9	0.098	41	24 (7.34)^**†**^	51	24 (7.67) ^**†**^	11	4 (2.44)
16.0–17.9	0.426	4	1 (0.31)	8	2 (0.64)	4	1 (0.61)
10.0–17.9		**150**	**85 (25.99)**^†^	**140**	**64 (20.45)**	**85**	**29 (17.68)**
**Girls**							
10.0–12.9	0.115	1	1 (0.31)^**†**^	0	0 (0.00)	27	13 (7.93)
13.0–13.9	0.078	45	31 (9.48)	59	19 (6.07)	17	9 (5.49)
14.0–14.9	0.139	64	41 (12.54)^⁑†^	57	20 (6.39)	15	7 (4.27)
15.0–15.9	0.174	53	27 (8.26)^**†**^	52	23 (7.35)^**†**^	15	5 (3.05)
16.0–17.9	0.508	14	8 (2.45)^⁑^	5	0 (0.00)	4	2 (1.22)
10.0–17.9		**177**	**108 (33.03)**⁑†	**173**	**62 (19.81)**	**78**	**36 (21.95)**
Overall		**327**	**193 (59.02)**^⁑†^	**313**	**126 (40.26)**	**164**	**65 (39.63)**
**Extreme elevations (KIM-1 > 97.5th quantile of RI)**							
Boys		150	5 (1.53)	140	2 (0.64)	85	2 (1.22)
Girls		177	10 (3.06)^⁑^	173	1 (0.32)	78	1 (0.61)
Overall		327	15 (4.59)^⁑^	313	3 (0.96)	164	3 (1.83)

The reference interval and the median KIM-1 of each age stratum are given for each age group.

The number of children in each age stratum with urinary KIM-1 levels above the RI (50th quantile at 90% CI) given in the previous study^35^, for that particular age group is given as N along with its percentage with respect to the total size of the respective study group, endemic, emerging, or non-endemic. Similarly, the number of children with urinary KIM-1 levels above the 97.5th quantile of RI are given as extreme elevations. Comparison of proportions of children above the RI is expressed according to Chi-squared test and for the age strata with (N < 20), Fisher's exact test was used for comparison. ^⁑^Denotes significance compared to the counterparts of same age in emerging group (*p* < 0.05) and ^**†**^denotes significance compared to the counterparts of same age in non-endemic group.

*RI* reference intervals of biomarkers, *N*_*T*_ total number of children in an age stratum within the study group, *KIM-1:* kidney injury molecule-1.

**Table 4:** The incidence of elevated expression of of urinary NGAL in children from CKDu endemic, emerging and non-endemic regions in Sri Lanka.

Age group	RI^35^	Endemic		Emerging		Non-endemic	
		N_T_	NGAL > RI N (%)	N_T_	NGAL > RI N (%)	N_T_	NGAL > RI N (%)
**Boys**							
10.0–12.9	3.748	4	3 (0.92)^**†**^	0	0 (0.00)	31	14 (8.54)
13.0–13.9	2.966	55	34 (10.40)^⁑^	39	7 (2.24)^**†**^	22	16 (9.76)
14.0–14.9	3.299	46	22 (6.73)	42	20 (6.39)	18	12 (7.32)
15.0–15.9	3.278	41	11 (3.36)^⁑^	51	31 (9.90)	11	8 (4.88)
16.0–17.9	4.851	4	1 (0.31)	8	5 (1.60)	4	3 (1.83)
**10.0**–**17.9**		**150**	**71 (21.71)**^**†**^	**140**	**63 (20.13)**^**†**^	**86**	**53 (32.32)**
**Girls**							
10.0–12.9	3.146	1	1 (0.31)^†^	0	0 (0.00)	27	10 (6.10)
13.0–13.9	2.085	45	34 (10.40)^⁑^	59	13 (4.15)	17	9 (5.49)
14.0–14.9	3.128	64	34 (10.40)^†^	57	27 (8.63)	15	8 (4.88)
15.0–15.9	2.984	53	19 (5.81)^⁑^	52	33 (10.54)^†^	15	7 (4.27)
16.0–17.9	3.496	14	6 (1.83)	5	3 (0.96)	4	3 (1.83)
**10.0**–**17.9**		**177**	**94 (28.75)**	**173**	**76 (24.28)**	**78**	**37 (22.56)**
**Overall**		**327**	**165 (50.46)**	**313**	**139 (44.41)**	**164**	**90 (54.87)**
**Extreme elevations (NGAL > 97.5th quantile of RI)**							
Boys		150	4 (1.22)	140	3 (0.96)	86	0 (0)
Girls		177	5 (1.55)	173	3(0.96)	78	0 (0)
Overall		327	9 (2.73)^†^	313	6 (1.92)	174	0 (0)

The reference interval and the median NGAL of each age stratum are given for each age group.

The number of children in each age stratum with urinary NGAL levels above the RI (50th quantile at 90% CI) given in the previous study^35^, for that particular age group is given as N along with its percentage with respect to the total size of the respective study group, endemic, emerging or non-endemic. Similarly, the number of children with urinary KIM-1 levels above the 97.5th quantile of RI are given as extreme elevations. Comparison of proportions of children above the RI is expressed according to Chi-squared test and for the age strata with (N < 20), Fisher's exact test was used for comparison. ^⁑^Denotes significance compared to the counterparts of same age in emerging group (*p* < 0.05) and ^†^denotes significance compared to the counterparts of same age in non-endemic group.

*RI* reference intervals of biomarkers, *N*_*T*_ total number of children in an age stratum within the study group, *NGAL:* neutrophil gelatinase-associated lipocalin.

Particularly in CKDu endemic regions, boys and girls in most of the age strata showed significantly higher incidence of elevated urinary KIM-1 levels above the reference intervals reported in the previous study^35^ compared to the children in CKDu non-endemic regions. When considering the entire age range, boys and girls in CKDu endemic regions reported significantly higher incidence of elevated KIM-1 levels. Further, irrespective of gender and age, the prevalence of elevated KIM-1 levels among the children in CKDu endemic regions (59.02%) was significantly higher (*p* < 0.01) than that in both CKDu emerging and non-endemic regions.

Urinary NGAL levels of girls and boys also showed substantial variations among the three study groups at several age strata. Boys in nonendemic regions reported significantly high (*p* < 0.05) urinary NGAL levels compared to the boys in the endemic group, where girls reported no significant difference. However, overall analysis showed no significant differences among the proportions of participants with NGAL levels above the RI, in the three study groups (Table 4).

#### Incidence of albuminuria

Potential manifestations in renal function were assessed with urinary ACR level, categorized into two groups as low-risk non-albuminuria (ACR < 30 mg/g) and high-risk albuminuria (ACR > 30 mg/g). The number of participants in each category are shown in Table 5.

**Table 5:** ACR-based stratification of the potential risk of renal injury and the related incidence in children from CKDu endemic, emerging, and non-endemic regions.

Residential CKDu prevalence	Gender	N	Albuminuric (ACR ≥ 30 mg/g) Incidence (%)	Nonalbuminuric (ACR < 30 mg/g) Incidence (%)
Endemic (N = 327)	Boys	150	2 (0.6)	148 (45.3)
	Girls	177	4 (1.2)	173 (52.9)
Emerging (N = 313)	Boys	140	1 (0.3)	139 (44.4)
	Girls	173	3 (1.0)	170 (54.3)
Non-endemic (N = 164)	Boys	86	1 (0.6)	85 (51.8)
	Girls	78	1 (0.6)	77 (47.0)

The incidence is given as the number and percent of the total size of the study group in the respective region; CKDu endemic, emerging or non-endemic.

*ACR* albumin creatinine ratio.

The majority of the children in CKDu endemic, emerging, and non-endemic regions were in the low-risk, no albuminuria category. The prevalence of potential manifestations in renal function, as indicated by albuminuria was 1.8%, 1.3%, and 1.2% in CKDu endemic, emerging and non-endemic regions respectively. The proportions showed no significant difference (*p* > 0.05) across the locations.

#### Biomarker levels in the participants with and without albuminuria

Biomarker levels in children with and without albuminuria are presented in Table 6. Participants in CKDu endemic areas without albuminuria reported significantly higher urinary KIM-1 levels (*p* < 0.01) compared to the other two groups. However, NGAL levels in nonalbuminuric children showed no significant difference between participants from endemic and non-endemic regions. The number of albuminuric children was very low in all groups.

**Table 6:** Biomarker distribution of children with and without albuminuria.

Residential CKDu prevalence	Biomarker levels (ng/mgCr) median (IQR)					
	Albuminuric children			Nonalbuminuric children		
	N	KIM-1	NGAL	N	KIM-1	NGAL
Endemic (N = 327)	6	0.534 (0.335–1.357)	4.991 (3.407–18.23)	321	0.187 (0.025–0.388)	2.945 (1.801–5.506)
Emerging (N = 313)	4	0.089 (0.023–0.128)	2.447 (1.995–2.745)	309	0.080^⁑^ (0.004–0.226)	2.518 (1.330–4.886)
Non-endemic (N = 164)	2	0.774 (0.003–1.545)	2.311 (2.258–2.364)	162	0.040 ^**†**^ (0.001–0.240)	3.366 (1.728–5.170)
Overall	12	0.331* (0.087–0.883)	3.028 (2.284–5.744)	792	0.112 (0.003–0.306)	2.961 (1.548–5.149)

Albuminuria was defined as urinary ACR ≥ 30 mg/g. Statistical comparison of biomarkers in nonalbuminuric children among the three prevalence categories is expressed as implied by Kruskal–Wallis test followed by Dunn’s multiple comparison test. Comparison of overall biomarker levels between albuminuric and nonalbuminuric groups is given according to Mann–Whitney U test. ^⁑^Significant compared to emerging group (p = 0.0002), ^†^significant compared to non-endemic group (p = 0.0001), *significant compared to the nonalbuminuric group (p = 0.032).

*KIM-1* kidney injury molecule-1, *NGAL* neutrophil gelatinase associated lipocalin, *ACR* albumin creatinine ratio.

When analyzed as a single group, participants with albuminuria reported significantly higher (*p* = 0.032) urinary KIM-1 level compared to the participants with no albuminuria. Here, urinary NGAL level also showed no significant difference between the albuminuric and nonalbuminuric groups (*p* = 0.334).

Within the context of findings, urinary KIM-1 expression demonstrated profound differences across the three regions showing elevated expression in participants with increasing prevalence of CKDu in residential areas. Also, urinary KIM-1 showed elevated expression in albuminuric children. On the contrary, urinary NGAL did not demonstrate noteworthy variations with residential CKDu prevalence or albuminuria. As such, KIM-1 appears to be a more sensitive marker that indicates potentially low-grade renal injury in children, where the sensitivity of urinary ACR becomes inadequate. Hence, KIM-1 may be preferentially used over NGAL and ACR as a marker to characterize early renal injury among children.

## Discussion

CKDu is an emerging threat to public health across several tropical countries including Sri Lanka, but studies remain primarily focused in adult populations. Here we focused on renal function in a pediatric population in Sri Lanka and report the first study to use KIM-1 and NGAL to assess pediatric renal health. This is an extension of our previous study for defining reference intervals for urinary KIM-1 and NGAL for the pediatric population in Sri Lanka^35^. The main objective of the present study was to assess the utility of KIM-1 and NGAL to identify early renal injury or renal abnormalities in a pediatric population in CKDu endemic, emerging and non-endemic areas in Sri Lanka. A large sample size with wide age distribution is the main strength of our study, and longitudinal observation of renal function in our study population remains a significant next step.

Our findings demonstrate significantly higher expression of KIM-1 in the participants from CKDu endemic regions compared to those from CKDu emerging and non-endemic regions. This is in accordance with previous studies based on albumin and creatinine indicating adverse renal health outcomes in pediatric populations in CKDu impacted regions in Sri Lanka^8,36^.

Further, girls generally reported higher KIM-1 expression than the boys in the same group and the difference was significant in CKDu endemic and emerging regions. In healthy individuals, KIM-1 may appear in urine at very low levels and elevation of KIM-1 expression may indicate renal insult^21,25^. However, this difference cannot be reliably attributed to renal injury in the children with the available data. Nonetheless, the notably high expression of urinary KIM-1, particularly in children from CKDu endemic regions, is indicative of low-grade early renal damage.

In comparison to KIM-1, urinary NGAL levels in the study participants did not demonstrate significant differences across endemic emerging and non-endemic regions. In gender-wise analysis, urinary NGAL expression showed no significant difference among boys in the three study groups. Girls in CKDu endemic regions showed significantly elevated expression of NGAL compared to the girls in emerging regions, but not with the girls in non-endemic regions. The expression of NGAL in urine tends to increase markedly above the normal levels, in cases of tubular injury. However, based on the variation of NGAL among the three study groups in our study, it is difficult to produce a comparative interpretation on renal health of the current study participants.

Urinary ACR in children showed no significant variation among the three study groups. Even in gender-wise analysis, no significant variations were noted. Further, no significant differences of ACR were noted among the boys and girls within the same group. In contrast to the novel biomarkers, urinary ACR appears to be less sensitive in indicating minor alterations in renal function. Based on elevated ACR (ACR ≥ 30 mg/g), we identified albuminuria in several participants; in total 12 children, 6 (1.8%) from endemic regions, 4 (1.3%) from emerging regions, and 2 (1.2%) from non-endemic regions, were reported with albuminuria. However, these proportions were not significantly different across the locations. Importantly, children living in the endemic areas with no albuminuria had significantly high urinary KIM-1 levels, where NGAL showed no significant variations. As such, we posit that KIM-1 may have the ability to detect sub-clinical renal injury in children with normal urinary microalbumin levels.

High Urinary KIM-1 levels were observed in children with albuminuria in both endemic and emerging regions, compared to the children in non-endemic areas. If children have albuminuria and elevated KIM-1, the probability of renal injury is higher. However, as the number of children with albuminuria was low (N = 12) compared to the total study population (N = 792), the predictive value of these biomarkers is unclear. In children, histologically normal, self-limiting orthostatic proteinuria also needs to be considered. Hence the statistical comparison between the two groups may not be satisfactory.

Although CKDu is predominant among adult communities, two previous studies conducted in CKDu-affected regions demonstrated renal injury among younger individuals. In a community study conducted in the year 2003 in Medawachchiya, an endemic area in the NCP in Sri Lanka, 7.7% (2/26) of the participants with CKD stages 3–5 were below 20 years^36^. In a recent study in the NCP with 2880 school children between 5–11 years of age, 8.7% had albuminuria and some had low eGFR^8^. In our study, the prevalence of albuminuria was less than 2% in endemic and non-endemic areas. Similar findings were observed from CKDu hotspots in Mesoamerica. In a study of 200 school students (12–18 years) from CKDu affected areas in Nicaragua, 16 (8%) had albuminuria. The median (IQR) NGAL levels in boys and girls were 25.7 (15.6–49.6) ng/ mgCr and 7.0 (4.2–10.9) ng/ mgCr respectively, with participants from high-risk areas reporting higher NGAL levels^37^. However, they did not measure KIM-1 in the urine and found an association with dysuria with high NGAL levels. Further, urinary NGAL levels in girls were higher than those in boys. In a similar study with 210 school students (7–17 years of age) from areas with high risk of CKDu in Nicaragua 8 (4%) had low eGFR below 90 mL/min/1.73 m^2^. The median (IQR) KIM-1 levels were 0.713 (0.388–1.086) ng/mgCr and, 1.058 (0.627–1.628) ng/mgCr, and the median NGAL levels were 4.9 (2.7–7.6) and 20.9 (10.04–40.8) for girls and boys respectively^9^. Girls had high urinary biomarker (KIM-1 and NGAL) levels, and dysuria was an associated symptom. The reported levels of KIM-1 and NGAL in Mesoamerican pediatric population, appear consistent with the biomarker levels observed in the present study. However, in comparison to these two Mesoamerican studies, we noted several distinct similarities, as well as differences in biomarker distribution patterns in our study participants in Sri Lanka. In both above studies, KIM-1 and NGAL levels in girls were found to be significantly high compared to the boys in the same age range. Being consistent with their findings, urinary KIM-1 levels in girls were significantly higher than KIM-1 levels of boys, in our study. In contrast, we noted significantly low urinary NGAL level in girls compared to the boys, in CKDu non-endemic regions. In CKDu endemic and emerging regions, urinary NGAL level did not show significant difference between boys and girls. However, climatic and sociodemographic determinants in Sri Lanka are distinctly diverse from those in Mesoamerica. Further, in comparison to Mesoamerican children, the degree of exposure of children to potential risk factors of CKDu appears to be less intense for the children in Sri Lanka. Additionally, a wide spectrum of risk factors related to environment, lifestyle, genetics and food and water contamination are associated with renal outcomes such as CKD/CKDu and renal injury. Hence, renal outcomes of the children in Sri Lanka may not be comparable with those of the children in Mesoamerica. Further, within the context of our investigation, the findings are not sufficient to interpret the observed gender differences of biomarker expression in children. Even in Mesoamerica, this observation still remains unexplained.

Defining benchmark values for KIM-1 and NGAL to predict renal injury enhances the prognostic value of these biomarkers, especially for CKDu impacted regions. The number of participants with the predictive renal outcome (elevated ACR above 30 mg/g) was low, leading to low accuracy in the classifier model in ROC analysis. Hence, future studies of participants with high ACR levels may yield better accuracy. In addition, the present study does not assess serum creatinine and eGFR, due to difficulty in obtaining consent for venesection in children. As this is likely a common challenge globally, improving the diagnostic probability based on urinary markers remains a high priority.

Our data also lend insights into the current discussion on aetiological factors associated with CKDu, including nephrotoxic environmental contaminants, fluoride in water, hard water, and exposure to heat stress/ dehydration^38–40^. Heat stress can lead to a cascade of biochemical events in the body leading to alterations in renal function and is an important hypothesis to consider^41^. In our survey during the establishment of study groups, we quantified hydration levels and amount of time spent farming per year (recall data), which suggested no difference in these two variables across the study group. Our data suggested that children are less likely to be exposed to farming associated heat stress and develop dehydration, compared to adults. However, exposure to the environmental contaminants remains high for children and adults in the areas with a high prevalence of CINAC in Sri Lanka due to drinking water contamination^4^; this study also showed that KIM-1 is induced following exposure to glyphosate in in vivo studies. Accordingly, the current data on KIM-1 supports a stronger role for chemical exposure induced renal injury early in life, further studies are needed to delineate the roles of heat stress and chemical contaminants on elevated KIM-1 in pediatric populations in Sri Lanka. Such studies will need to also develop a cohort completely outside of the dry zone, since evidence of disease emergence is reported outside the current endemic region boundaries^5^.

Adults populations have received extensive attention when screening for chronic kidney disease^42,43^. The absence of consensus on interventions has made screening for kidney disease in children less of a priority in many countries, although annual renal health screening is mandatory for school children in Japan, Taiwan, and Korea^44^. Identification of susceptibilities of renal diseases among school children as early as possible is vital to arrest the progression of renal diseases. Particularly, early stage renal diseases or low grade renal injury is often asymptomatic and nonproteinuric^12^, and the conventional diagnostic markers such as serum creatinine and albuminuria are not sensitive and specific enough in the detection of such nephropathies^32^. Hence, integration of emerging biomarkers with enhanced sensitivity and specificity such as KIM-1 into clinical practice along with routine renal health checks may contribute to develop effective management strategies against pediatric renal diseases.

## Conclusion

Our study shows that pediatric urinary KIM-1 expression increased with increasing CKDu burden in residential area, even in the absence of albuminuria. Particularly, KIM-1 is a molecule that may express at very low levels in healthy individuals and it is expressed at increased levels in case of renal injury. Hence, the expression of urinary KIM-1, particularly in children from CKDu endemic regions at significantly higher levels, may provide important indications on low-grade early renal injury or renal abnormalities. However, further studies including longitudinal analyses are necessary to draw strong conclusions on the renal health of the children in these regions. On the contrary, urinary NGAL in children did not demonstrate noteworthy variations with residential CKDu prevalence or albuminuria. Accordingly, KIM-1 appears to be a more sensitive marker, where the sensitivity of urinary ACR becomes inadequate. Early renal injury may lead to the development of CKD^45^, hence periodical monitoring of the renal function of children is likely to be critical in preventing the development of CKD. An increased sample size covering a larger area in CKDu endemic and non-endemic regions will contribute to more precise conclusions on the risk prediction leading to effective interventions against renal diseases.

## Data Availability

The datasets generated during and/or analyzed during the current study are not publicly available due restrictions under the approval of the ethics review board, but are available from the corresponding author on reasonable request.
